# RNA m^6^A meets transposable elements and chromatin

**DOI:** 10.1007/s13238-021-00859-2

**Published:** 2021-07-13

**Authors:** Chenxi He, Fei Lan

**Affiliations:** Shanghai Key Laboratory of Medical Epigenetics, International Laboratory of Medical Epigenetics and Metabolism, Ministry of Science and Technology, Institutes of Biomedical Sciences, Fudan University, and Key Laboratory of Carcinogenesis and Cancer Invasion, Ministry of Education, Liver Cancer Institute, Zhongshan Hospital, Fudan University, Shanghai, China

*N*^6^-methyladenosine (m^6^A) is the most abundant internal chemical mark in eukaryotic messenger RNAs (mRNAs), regulating various processes in the life cycle of mRNA including splicing, nuclear export, degradation and translation (reviewed in (Shi et al., 2019)). For decades, m^6^A has also been identified in non-coding RNAs including long non-coding RNAs (lncRNAs), small nuclear RNAs (snRNAs) and ribosomal RNAs (rRNAs) (Epstein et al., 1980; Maden, 1986; Dominissini et al., 2012). Recently, several studies from different groups found that m^6^A occurred in chromatin associated regulatory RNAs (carRNAs), including promoter-associated RNA (paRNA), enhancer RNA (eRNA) and RNA transcribed from transposable elements (repeat RNA), which in turn influenced chromatin environment and transcription (Li et al., 2020; Liu et al., 2020, 2021; Chen et al., 2021; Xu et al., 2021), through previously under-appreciated crosstalks between the m^6^A modification in RNA and epigenetics modifications in chromatin (Fig. 1). Since the majority of these studies focused on the regulation of the transposable elements (TEs), especially in the context of repressive chromatin, the main part of this commentary will center on m^6^A and transcription silencing.

**Figure 1:**
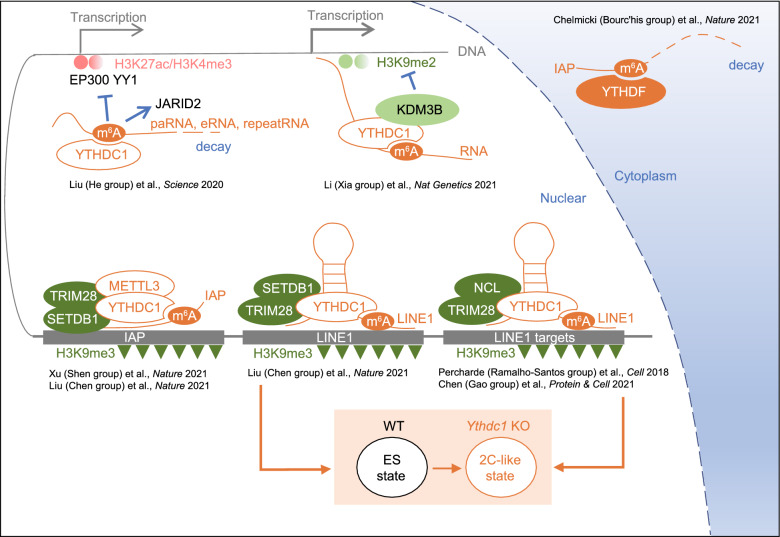
Schematic of m^6^A regulating chromatin environment

Accumulating evidence shows that RNA is extensively involved in transcription and epigenetics regulation, exemplified by the well-studied lncRNA XIST-mediated gene silencing on X chromosome during X-inactivation. Intriguingly, m^6^A modification was found in XIST RNA mediating its transcriptional repression function (Patil et al., 2016). In 2019, Xiao et al. systematically investigated the genome-wide chromatin occupation of dozens of RNA-binding proteins (RBPs) in HepG2 and K562, raising the concept that “transcription and co-transcriptional RNA processing might not simply be spatially and temporally co-incident events but could be more mechanistically integrate” (Li and Fu, 2019; Xiao et al., 2019). However, how the regulatory RNA molecules are regulated by m^6^A and how the modified RNA interplay with the epigenetics regulation in the chromatin context remain largely unclear until several recent studies mentioned above.

## m^6^A GUIDES HISTONE MODIFICATION IN ACTIVE CHROMATIN REGIONS

Although co-transcriptional m^6^A deposition in nascent transcripts by METTL3-METTL14 complex has been reported (Barbieri et al., 2017; Knuckles et al., 2017; Slobodin et al., 2017; Huang et al., 2019), whether the incorporated m^6^A modification may in turn affect local transcription or chromatin environment remains overlooked. The term of carRNA mentioned above was initially used in Liu’s study (Chuan He’s group), in which the authors showed that YTHDC1 facilitated the decay of a subset of m^6^A-modified carRNAs, including paRNA and eRNA (Liu et al., 2020). Loss of METTL3-mediated m^6^A promoted carRNA stability and transcription rate as well as local chromatin accessibility in mouse embryonic stem cells (mESCs), such processes were accompanied by elevated active histone marks of H3K4me3 and H3K27ac. The authors further showed that the global binding of EP300 and YY1 increased, while JARID2, a component of PRC2 complex (polycomb repression complex 2) in mESCs, decreased upon loss of METTL3 (Fig. 1, upper left). In contrast, another study from Li et al., showed that m^6^A in nascent transcript promoted gene expression through a mechanism that also involved YTHDC1 which recruited KDM3B to remove the repressive histone mark of H3K9me2 in mESCs (Fig. 1, upper middle) (Li et al., 2020). The differences in the findings might be due to different types of carRNAs and target genes studied, and the exact mechanism and function of m^6^A involved in the regulation of active chromatin required more investigation.

## m^6^A REGULATES REPRESSIVE CHROMATIN ENVIRONMENT OF TRANSPOSABLE ELEMENTS

TEs compromise a substantial proportion of mammalian genome, whose silencing is crucial for genome integrity. TEs could be classified into retrotransposons and DNA transposons by their transposition intermediate. Particularly, the retrotransposons include long interspersed nuclear elements (LINEs), short interspersed nuclear elements (SINEs) and long terminal repeat elements (LTRs) (Wicker et al., 2007; Chuong et al., 2017). Endogenous retrovirus (ERVs) is the predominant superfamily among LTRs, and is mainly composed of ERV1, ERVK as well as ERVL and MaLR. Although these elements are used to be thought as “junk sequences”, emerging evidence shows that they also take part in the regulation of host genome (Chuong et al., 2017).

In addition to promoter-associated and enhancer RNAs, Liu et al., (He group) also showed elevated stability and transcription rate of LINE1, an important type of repeat RNAs, upon METTL3 depletion. Consistently, Chelmicki et al. showed that both rapid or prolonged depletion of METTL3 and METTL14 in mESCs led to increased level of intracisternal A-particles (IAPs), a highly active subtype of ERVK in mouse genome (Chelmicki et al., 2021). The authors demonstrated that the m^6^A methylation in IAP transcripts could be recognized by YTHDF family proteins and resulted in decreased stability of the modified IAP transcripts (Fig. 1, upper right). While rapid depletion of both METTL3 and METTL14 greatly stabilized IAP transcripts, chromatin modifications including H3K4me3, H3K27ac and H3K9me3 were not changed, which was different from what happened to LINE1 in Liu’s study (He group). Whether these chromatin marks could be altered upon sustained METTL3/14 loss was not investigated in this study.

Other groups turned to another direction and found the connection between m^6^A and repressive histone marks, such as H3K9me3 and H4K20me3. The study by Xu et al. observed elevated IAP transcripts in *Mettl3* KO mESCs as well (Xu et al., 2021). However, in contrast to Chelmicki’s study, Xu et al. showed that METTL3 loss didn’t alter the stability of IAP transcripts, but significantly reduced the heterochromatin marks including H3K9me3 and H4K20me3 over the IAP genomic loci hence led to increased transcription. Importantly, Xu et al. further demonstrated that the genomic loci of IAP were bound by METTL3, and the nascent IAP transcripts were methylated by METTL3 and recognized by the m^6^A reader, YTHDC1, whose interaction with METTL3 reinforced this positive feedback loop. Importantly, such mechanism facilitated the recruitment of the H3K9me3 methyltransferase SETDB1 and its cofactor TRIM28 through the interaction with METTL3 to establish transcriptionally repressive environment covering the IAP genomic loci (Fig. 1, lower left).

## m^6^A MODIFIED LINE1 IS IMPORTANT FOR 2C GENE SUPPRESSION AND MESC IDENTITY

In the same issue of Nature, Liu et al. (Jiekai Chen’s group) also reported elevated level of IAP transcripts in *Ythdc1* conditional knockout (*Ythdc1* cKO, referred to *Ythdc1*^flox/flox^ expressing CreERT upon 4OHT treatment here and afterwards) mESCs and *Mettl3* KO mESCs (Liu et al., 2021). They proposed a similar mechanism as Xu et al. that the m^6^A modified IAP transcripts acted in the chromatin environment where they were generated and YTHDC1 was recruited through the recognition of the m^6^A (Fig. 1, lower left). Moreover, they again found the interaction between YTHDC1 and SETDB1, and such interaction also connected m^6^A regulation to the heterochromatin formation. Importantly, in addition to IAP, Liu et al. (Chen group) found that LINE1 was m^6^A modified and this mechanism also played a role in suppressing LINE1, which was known to bind and recruit TRIM28 to repress the key 2C transcriptional factor DUX maintaining the mESC identity by keeping the 2C (2-cell embryo) genes silent (Percharde et al., 2018).

Interestingly, Liu et al. (Chen group) further found that *Ythdc1* cKO and *Mettl3* KO mESCs indeed developed a 2C-like transcriptome with several 2C specific marks including *Zscan4* family genes, *Dux* and MERVL significantly de-repressed. These de-repressed 2C genes were mainly activated by the key factor DUX, as deletion of *Dux* locus completely blocked the 2C gene activation. Importantly, DUX silencing required YTHDC1 recognition of m^6^A as only the wildtype but not the m^6^A-binding mutant YTHDC1 could restore the DUX repression in *Ythdc1* cKO. Based on these findings, the authors speculated that it was the m^6^A modified LINE1 gathering YTHDC1 and SETDB1/TRIM28 together to suppress DUX transcription. Of note, *Ythdc1* cKO mESCs not only showed 2C like transcriptome, but were also capable of incorporating into extra-embryonic tissues by chimera assay in E4.5 embryos, although with severe defects in proliferation hence could not survive to E6.5. Using a milder knock-down approach, the authors were able to observe the incorporated *Ythdc1*-knockdown cells in both embryonic and extra-embryonic tissues by E6.5. Since only zygote and 2-cell embryo can develop to both embryonic and extra-embryonic tissues in mice, these findings demonstrated that though not fully competent as true 2C cells, *Ythdc1* cKO mESCs showed certain 2C features both transcriptionally and functionally (Fig. 1, lower middle).

In the recent issue of Protein & Cell, similar to Liu’s findings (Chen group), Chen et al. independently demonstrated that the *Ythdc1* cKO mESCs resembled a 2C-like state transcriptionally, including the de-repression of *Zscan4* family genes, *Dux*, MERVL and MMETn, again underlining the importance of YTHDC1 in self-renewal and maintenance of the mESCs state by keeping the 2C genes transcriptionally silent (Chen et al., 2021). Mechanistically, as LINE1 transcripts were previously reported as a scaffold for recruiting NUCLEOLIN and TRIM28 to repress 2C gene transcription (Percharde et al., 2018), the authors here further demonstrated that the YTHDC1 could bind m^6^A-modified LINE1 and interact with NUCLEOLIN-TRIM28 repressive complex, thus YTHDC1 and m^6^A took part in the LINE1-NUCLEOLIN-TRIM28 regulation axis, linking the m^6^A modification to histone H3K9me3 deposition at the LINE1-targeted loci (Fig. 1, lower right).

Different from Liu’s study (Chen group), Chen et al. found that the 2C genes or retrotransposons would not be activated by the depletion of METTL3, indicating a METTL3-independent function of YTHDC1. Interestingly, the authors further reported the m^6^A modification in LINE1 could be classified to METTL3-sensitive and METTL3-insensitive types according to the m^6^A-seq data in WT and *Mettl3* KO mES from Liu et al. (He group). The METTL3-sensitive m^6^A peaks contained classic METTL3 motif “RRACH”, while METTL3-insensitive m^6^A peaks were enriched for “ABAG” motif which was similar to the substrate motif recognized by METTL16, another RNA m^6^A methyltransferase known to regulate RNA splicing and sense cellular S-adenosine-L-Methionine (Pendleton et al., 2017; Mendel et al., 2021). Given that METTL16 deletion resulted in a greater reduction of YTHDC1 binding to LINE1 transcripts reported in Chen’s study, whether METTL16 was indeed responsible for the “ABAG” m^6^A modification in LINE1 and played a role in LINE1 transcriptional or post-transcriptional regulation should be an important question for future studies.

Functionally, Chen’s work found that the *Ythdc1* cKO mESCs had deficiencies in proliferation and showed abnormality in differentiation. Unlike the chimeric assay performed at earlier developmental time in Liu’s study (Chen group), Chen et al. only checked at E14.5 and failed to observe any incorporated *Ythdc1* cKO cells, likely due to proliferation failure. They also found that most *Ythch1* KO embryos died before E6.5 and even failed to hatch out of the zona pellucida at E4.5 when the maternal YTHDC1 was removed. As the *Mettl3* KO embryos were previously reported to be able to develop till E7.5 (Geula et al., 2015), they speculated that YTHDC1 had METTL3 independent function. However, Kasovitz et al. previously showed that *Ythdc1* KO embryos could survive till E9.5 (Kasowitz et al., 2018), so to what extent the function of YTHDC1 relied on METTL3 needed more investigation.

## LIMITATIONS AND PERSPECTIVES

Emerging evidence shows that the silencing of TEs is precisely controlled and plays a role in genome regulation (Chuong et al., 2017). These recent m^6^A studies raise an interesting question of how TEs and host genomes are co-evolved. Both Xu’s study and Liu’s study (Chen group) mentioned an orthologous regulation of H3K9 methylation in *S*. *pombe* involving a YTH-domain-containing protein Mmi1 but is independent of m^6^A (Zofall et al., 2012; Wang et al., 2016). Of note, while paralogs of METTL3 and METTL14 are absent from *S*. *pombe* and *C*. *elegans*, they do exit in *S*. *cerevisiae* and *D*. *melanogaste*r (Balacco and Soller, 2019). How the host takes the advantage of m^6^A regulation in different classes of transcripts derived from ancient viral genome and evolve m^6^A “writers” and “readers” to control TE expression and genome integrity? One recent study found that the m^6^A in the 5’UTR of LINE1 RNA would enhance LINE1 translation to produce more ORF1 encoded proteins in human cells, and the METTL3 motif, DRACH, was positively selected over time (Hwang et al., 2021). Here, Liu et al. (He group) and Chen et al. found that younger LINE1s were more enriched for m^6^A signals among the LINE1 family, indicating that RNA m^6^A may also play a suppressive role in the newly integrated retroviral sequences as a part of host defense system. A plausible hypothesis is that LINE1 mRNAs hijack the m^6^A for effective translation, while the host may take the advantage to suppress LINE1 transcriptionally.

With all the advances they made, some limitations existed in the studies discussed above. For instance, all studies have been conducted in mESC, thus what happens in more differentiated cells and in other species remains to be elusive. Of note, IAP is a subfamily of ERVK and absent from human genome. The findings in mESC invite speculation that similar regulation may be involved in repression HERVKs, an active type of primate-specific TE (Johnson, 2019). Regarding the DUX regulation, Liu et al. (Chen group) nicely showed that LINE1 could be modified by m^6^A, *Dux* locus was bound by LINE1, and m^6^A was important for DUX silencing in mESCs. However, the exact mechanism was not entirely clear. For example, whether the YTHDC1 and SETDB1 recruitment, and H3K9me3 deposition at *Dux* locus were impaired? On a separate note, besides SETDB1, H3K9me3 modification over LINE1 loci was also reported to be regulated by SUV39H1/2, another H3K9me3 methyltransferase capable of interacting with RNA (Matsui et al., 2010; Bulut-Karslioglu et al., 2014; Li and Fu, 2019). How LINE1 status is co-regulated by the different suppressive mechanisms is an interesting question.

There were also some controversial results reported in these studies. For instance, Chelmicki et al. and Xu et al. found that LINE1 RNA levels were decreased upon METTL3 depletion, while Liu et al. (He group), Liu et al. (Chen group) and Chen et al. showed the opposite. In addition, Liu et al. (Chen group) found the *Mettl3* KO also induced 2C genes, while Chen et al. didn’t. The discrepancies might be explained by variations in experimental conditions or the different approaches to generate genetic knockout lines.

Overall, these recent findings are of importance, as they showed that the m^6^A modified transcripts derived from TEs could act both *in cis* and *in trans* to regulate the chromatin environment. In other words, the “RNA m^6^A-histone modification” node could involve different recruitment mechanisms and RNA types to exert its regulatory function in a context dependent manner. Regarding the exact molecular mechanism, it will be interesting to determine whether these chromatin associated transcripts are modified by m^6^A co-transcriptionally and crosstalk to histone modifications immediately after they are generated, or they receive the m^6^A modification in different compartments and then cycle back to their genomic loci through yet-to-be identified pathways. Another problem needs to be addressed is that how the YTHDC1-SETDB1/TRIM28-H3K9me3 mechanism distinguish different RNAs types. As m^6^A also exists in eRNA and mRNA derived from active chromatin, the mechanism has to be precisely targeted to heterochromatin. Nevertheless, with the establishment of the connection between the RNA m^6^A and histone modifications, these recent studies have opened a new revenue of m^6^A research, whether there are more uncovered crosstalking mechanisms in conjunction of RNA and epigenetics modifications calls for more future studies.

## DECLARATIONS

FL was supported by the National Key Research and Development program of China (2016YFA0101800 and 2018YFA0108700), the national Natural Science Foundation of China (31925010) and Shanghai Municipal Science and Technology Major Project (2017SHZDZX01).
